# Effectiveness of an Online Mindfulness-Based Intervention for Improving Mental Health in Undergraduate Students: A Randomized Clinical Trial

**DOI:** 10.3390/ijerph23070853

**Published:** 2026-06-30

**Authors:** Fabiane Penachiotti, Mirian Yamaguchi, Braulio Henrique Branco, Gabriela Alves, Felipe Rodrigues, Frederico Mariano, Adi Mana, Shifra Sagy, Rute Grossi-Milani

**Affiliations:** 1 Graduate Program in Health Promotion, Cesumar University, Maringá 87050-390, Brazil; mirianueda@gmail.com (M.Y.); braulio.branco@unicesumar.edu.br (B.H.B.); rute.milani@unicesumar.edu.br (R.G.-M.); 2 Cesumar Institute of Science, Technology and Innovation, Cesumar University, Maringá 87050-390, Brazil; 3 Department of Psychology, Cesumar University, Maringá 87050-390, Brazil; 4 Department of Biosciences, Federal University of Sao Paulo, São Paulo 11015-020, Brazil; 5 School of Behavioral Studies, Peres Academic Center, Rehovot 76120, Israel; 6 Martin Springer Center for Conflict Studies, Ben-Gurion University of the Negev, Beer Sheva 8410501, Israel

**Keywords:** anxiety, depression, mental health, mindfulness, resilience, sense of coherence, perceived stress, undergraduate students, higher education

## Abstract

**Highlights:**

**Public health relevance—How does this work relate to a public health issue?**

- Mental health problems among university students have become a growing global concern. Symptoms of anxiety, depression, and stress are increasingly prevalent in this population and can negatively affect academic performance, social functioning, quality of life, and future professional development.
- Online mindfulness-based interventions are accessible, scalable, and low-cost strategies that may expand access to mental health promotion and prevention initiatives for young adults.

**Public health significance—Why is this work of significance to public health?**

- This randomized controlled trial provides evidence that an online mindfulness-based intervention can improve mental health outcomes, including stress, anxiety, depression, psychological well-being, resilience, sense of coherence, and mindfulness among university students.
- The findings support the potential of remotely delivered mindfulness programs as preventive and health-promoting strategies that can strengthen psychological resources and complement existing mental health services.

**Public health implications—What are the key implications or messages for practitioners, policy makers and/or researchers in public health?**

- Online mindfulness interventions may be incorporated into university and community-based mental health promotion programs to increase access to evidence-based preventive strategies.
- The scalability and relatively low cost of digital interventions may help reduce barriers to mental health support and inform the development of population-level approaches for promoting well-being, particularly in resource-constrained settings.

**Abstract:**

This randomized clinical trial evaluated the effectiveness of an online mindfulness-based intervention in improving positive mental health and reducing symptoms of anxiety and depression among undergraduate students, in the context of rising global mental health challenges and limited access to care, particularly in low- and middle-income settings. Brazilian undergraduate pedagogy students enrolled in distance education were randomly assigned (1:1) to an eight-week Mindfulness-Based Program for Undergraduate Students or a control group. Primary outcomes included positive mental health, anxiety, and depression, while secondary outcomes were sense of coherence, resilience, mindfulness, and perceived stress. Analyses followed the intention-to-treat principle. A total of 317 students were randomly allocated to the intervention group (*n* = 157) or the control group (*n* = 160), and 215 (67.8%) completed the study, with comparable baseline characteristics across groups. Compared with controls, the intervention significantly improved positive mental health and reduced anxiety and depression (all *p* < 0.001), while also increasing sense of coherence, resilience, and mindfulness and reducing perceived stress (all *p* < 0.05). These findings suggest that scalable, low-cost, online mindfulness interventions can simultaneously enhance positive mental health and reduce psychological distress, supporting their potential as preventive mental health strategies in higher education settings.

## 1. Introduction

Higher education students represent a strategically important population for societal development, as they constitute a substantial proportion of the future workforce and contribute to social, economic, and scientific progress [1]. Universities play a central role not only in the acquisition of knowledge and professional competencies but also in the development of personal, social, and civic capacities that influence individuals’ future trajectories and contributions to society. The university years coincide with a critical developmental period in which important trajectories related to health, education, productivity, and social participation are established [2]. Experiences occurring during this stage may have lasting consequences for academic achievement, occupational opportunities, social integration, and long-term well-being [1]. Consequently, promoting mental health among university students is not only an educational priority but also a public health imperative, as investments in student well-being may generate benefits that extend beyond the individual level and contribute to healthier, more productive, and socially engaged communities [1].

The transition from adolescence to adulthood is characterized by profound psychological, social, and biological changes, a developmental stage often referred to as emerging adulthood [2]. During this period, individuals face important developmental tasks, including identity formation, increasing autonomy, academic achievement, and preparation for future professional roles. These transitions often occur simultaneously with exposure to new academic, social, and financial demands, which may increase vulnerability to psychological distress. Consistent with this developmental perspective, most common mental disorders emerge before the age of 25 [3]. Recent meta-analytic evidence indicates that approximately half of all lifetime mental disorders begin before mid-adolescence and the majority before young adulthood, highlighting the importance of prevention and early intervention during the university years [1,4].

Growing evidence indicates that mental health problems among university students constitute a major global public health concern. Data from the WHO World Mental Health International College Student Project, conducted across multiple countries, showed that approximately one-third of university students meet the criteria for at least one mental disorder [5]. Consistent findings from recent systematic reviews and meta-analyses have documented high rates of anxiety, depression, stress, and suicidal ideation across diverse educational, cultural, and socioeconomic contexts, highlighting the widespread nature of this challenge [6,7]. This pattern is also evident in Brazil, where a systematic review and meta-analysis estimated prevalence rates of approximately 38% for anxiety symptoms, 29% for depressive symptoms, and 9% for suicidal behavior among Brazilian undergraduate students [8]. Importantly, the burden of mental health problems among university students appears to be consistently higher than that observed in similarly aged individuals from the general population in several countries, suggesting that the university environment may expose students to unique stressors and vulnerabilities [5].

Beyond their high prevalence, mental health problems among university students have important academic, social, and long-term consequences. Mental health problems have been associated with poorer academic performance, lower academic engagement, impaired social functioning, reduced quality of life, and increased risk of university attrition [5,9]. Given that university students constitute a substantial proportion of the future workforce, the consequences of untreated mental health problems may extend beyond the educational setting and influence long-term health, productivity, and socioeconomic outcomes throughout adulthood [1]. Consequently, student mental health has become an increasing priority for higher education institutions worldwide [1].

Despite the growing burden of mental health problems, many university students do not seek professional help even when experiencing clinically significant symptoms, often due to barriers related to mental health literacy, stigma, accessibility, and service availability [10]. This treatment gap highlights the need for preventive and health-promoting approaches that are accessible, scalable, and capable of reaching large student populations [11,12]. Accordingly, international public health frameworks advocate the implementation of interventions that strengthen emotional and self-regulatory competencies while promoting psychological well-being across the lifespan [11,12].

Among the available approaches, mindfulness-based interventions (MBIs) have emerged as promising strategies for promoting mental health and preventing psychological disorders. Mindfulness is commonly defined as the capacity to intentionally attend to present-moment experiences with an attitude of openness, curiosity, and non-judgmental awareness [13]. Beyond its role in reducing psychological distress, mindfulness has also been consistently associated with greater psychological well-being, self-regulation, and optimal functioning [14]. Contemporary theoretical models conceptualize mindfulness as a self-regulatory process that influences mental health through multiple interconnected mechanisms. These mechanisms include enhanced attentional control, improved emotional regulation, reduced cognitive reactivity, and decreased engagement in maladaptive cognitive processes such as rumination and excessive worry [15,16]. More recent evidence suggests that mindfulness may also exert its effects through processes such as acceptance, decentering, and enhanced present-moment awareness, which facilitate adaptive responses to emotional experiences and contribute to improved well-being [17,18].

This perspective is consistent with the salutogenic model proposed by Antonovsky, which shifts the focus from the origins of disease to the factors that support health, adaptation, and well-being in the face of life stressors [19]. Rather than conceptualizing mental health solely as the absence of psychological symptoms, salutogenesis emphasizes the identification and strengthening of health assets that enable individuals to maintain and enhance well-being under challenging circumstances [19,20,21]. In this framework, resources such as positive mental health, resilience, and sense of coherence are considered important protective factors that may buffer the adverse effects of stress and contribute to healthier adaptation.

Positive mental health encompasses emotional, psychological, and social well-being and reflects the presence of positive functioning beyond the absence of mental illness [22]. Unlike traditional psychopathological indicators, positive mental health captures flourishing, optimal functioning, and the presence of psychological resources that enable individuals to thrive in everyday life. Resilience refers to the capacity to adapt successfully to adversity and recover from challenging life experiences, whereas sense of coherence reflects an individual’s ability to perceive life as comprehensible, manageable, and meaningful [19,23]. Individuals with a stronger sense of coherence are more likely to mobilize available resources and cope effectively with stressors, thereby supporting well-being and adaptation [19,20]. Evidence suggests that positive mental health, resilience, and sense of coherence are consistently associated with better mental health outcomes, greater well-being, and lower vulnerability to psychological distress [24,25]. Together, these resources can be understood as important health assets that support adaptation and psychological functioning in the face of adversity [20,21].

From a salutogenic perspective, mindfulness may contribute not only to reducing psychological distress but also to strengthening protective psychological resources. Evidence from systematic reviews and meta-analyses indicates that mindfulness-based interventions (MBIs) can reduce symptoms of anxiety, depression, and perceived stress while promoting psychological well-being [26,27,28,29]. In addition, MBIs have been associated with increases in resilience, self-compassion, and other protective psychological resources that may contribute to sustained improvements in mental health and adaptation [29,30,31].

Most mindfulness-based interventions evaluated in randomized controlled trials have adopted programs lasting approximately eight weeks, following the structure originally established by Mindfulness-Based Stress Reduction (MBSR) and subsequently incorporated into related protocols such as Mindfulness-Based Cognitive Therapy (MBCT) and Mindfulness-Based Health Promotion (MBHP). This format has accumulated substantial empirical support and remains the most widely investigated approach for improving psychological well-being and reducing symptoms of anxiety, depression, and stress [26,27,29]. The rationale for multi-session programs is that the cultivation of mindfulness involves the progressive development of attentional, emotional, and self-regulatory skills through repeated practice and integration into daily life. Consequently, evaluating standard-length interventions remains important, particularly when broader salutogenic outcomes, such as positive mental health, resilience, and sense of coherence, are considered.

At the same time, growing interest in scalability, feasibility, and participant engagement has stimulated the development of abbreviated mindfulness interventions. Studies conducted among university students have shown that programs ranging from four to six sessions, as well as brief online and app-based interventions, can produce meaningful improvements in stress, anxiety, well-being, and other psychological outcomes [32,33,34,35]. However, findings remain mixed regarding whether shorter formats achieve effects comparable to standard-length programs across all outcomes. While brief interventions may offer advantages in accessibility and adherence, longer programs may provide additional opportunities for the consolidation of mindfulness skills and the development of broader salutogenic resources. Consequently, the optimal duration and intensity of mindfulness-based interventions remain important questions for future research, particularly in university populations and large-scale public health applications.

More recently, online mindfulness-based interventions have received increasing attention as a potentially scalable approach to mental health promotion, particularly within university settings. By overcoming barriers related to accessibility, scheduling constraints, geographical distance, and the limited availability of mental health professionals, online delivery formats may expand the reach of evidence-based interventions to larger student populations [36]. Systematic reviews and meta-analyses support the effectiveness of online mindfulness-based interventions in improving mental health outcomes among university students [36]. However, important gaps remain regarding their implementation and evaluation across diverse cultural contexts, particularly in low- and middle-income countries. Furthermore, while numerous randomized controlled trials have demonstrated the effectiveness of mindfulness-based interventions in reducing anxiety, depression, and perceived stress, substantially fewer studies have simultaneously examined positive mental health and salutogenic resources such as resilience and sense of coherence. This gap is particularly evident in online interventions and in studies conducted in middle-income countries. Addressing this limitation is important because contemporary models of mental health emphasize not only the reduction in psychological distress but also the strengthening of psychological resources that support well-being, adaptation, and long-term mental health.

Therefore, the present study aimed to evaluate the effectiveness of an online mindfulness-based intervention on mental health and related psychological outcomes among Brazilian undergraduate students. Based on contemporary theoretical models, salutogenic principles, and previous empirical evidence, we hypothesized that participation in the intervention would reduce symptoms of anxiety and depression, enhance positive mental health, and strengthen key salutogenic resources, including mindfulness, resilience, and sense of coherence.

## 2. Materials and Methods

### 2.1. Study Design and Participants

This study is a randomized, parallel-group clinical trial conducted with undergraduate pedagogy students enrolled in a distance learning (DL) program in Brazil. The trial was registered in the Brazilian Registry of Clinical Trials (ReBEC) and followed the Consolidated Standards of Reporting Trials (CONSORT) guidelines [37]. The completed CONSORT checklist is provided as Supplementary File S1.

Participants were recruited voluntarily between 27 May and 20 June 2023, via the virtual learning environment of a Brazilian higher education institution with nationwide coverage. Invitations were made during online classes, and additional information, including access links to the mindfulness program (PMU), was disseminated through the institutional platform and by email. All participants provided electronic informed consent prior to enrollment.

Eligibility criteria included being aged 18 years or older, regularly enrolled in the DL program, and having access to the internet and digital devices required for participation. Students who reported severe psychiatric conditions or were undergoing intensive psychological or psychiatric treatment were excluded.

Participants allocated to the control group continued their usual academic activities without receiving the mindfulness intervention during the study period. After trial completion, they were offered access to the program.

### 2.2. Randomization and Allocation

Participants were randomly assigned in a 1:1 ratio to either the intervention group (Mindfulness-Based Program for Undergraduate Students—PMU) or the control group. Randomization was performed using a computer-generated sequence, ensuring allocation concealment.

Due to the nature of the intervention, participants were not blinded to group allocation. However, data analysis was conducted following the intention-to-treat principle to minimize bias.

To ensure blinding of outcome assessment, all data were collected online using the Qualtrics platform, and statistical analyses were conducted by an independent statistician.

### 2.3. Intervention

The intervention consisted of an adapted version of the Mindfulness-Based Health Promotion (MBHP) program [38]. While the original MBHP protocol comprised eight weekly in-person sessions of approximately two hours, the present study maintained the eight-week structure but adapted sessions to an online format delivered via videoconferencing, with a duration of approximately one hour to enhance feasibility and accessibility. The program was delivered to participants in the intervention group from 27 June 2023 to 15 August 2023. The control group, in turn, received the intervention between 5 September and 24 October 2023.

The program was delivered by a certified instructor with approximately eight years of experience in MBHP. The adapted program preserved the core components of MBHP, emphasizing both formal and informal mindfulness practices integrated into daily life. Participants were encouraged to cultivate present-moment awareness during meditation as well as in routine activities such as walking, studying, and interpersonal interactions.

After each session, participants received audio-guided practices and daily activity suggestions via WhatsApp, with an average recommended practice duration of 20 min per day.

Core techniques included mindfulness of breathing, body scan, mindful movement, mindful walking, and brief practices such as the 3 min breathing space. Psychoeducational components addressed concepts such as primary and secondary suffering, acceptance, compassion, and self-compassion.

The eight sessions followed a structured progression: (1) introduction to mindfulness and automatic pilot; (2) mindfulness of the body; (3) mindfulness in movement; (4) expanding awareness; (5) dealing with difficulties; (6) mindfulness and silence; (7) compassion; and (8) mindfulness for everyday life.

Participants who were unable to attend a live session were provided access to a recording of the session. However, access to or viewing of the recordings was not systematically monitored. Therefore, adherence measures were based exclusively on attendance at live sessions.

Safety procedures included monitoring for adverse effects through weekly check-ins and availability of psychological support. No adverse events or intervention-related complications were reported.

The program was offered free of charge, and participation was voluntary. No financial incentives were provided; however, small engagement strategies (e.g., book raffles) were used to enhance adherence. Participants who attended at least four sessions and completed both assessments received a certificate of completion.

### 2.4. Outcomes

Primary outcomes were anxiety, depression, and mental health, assessed using the Generalized Anxiety Disorder-7 (GAD-7), Patient Health Questionnaire-9 (PHQ-9), and Mental Health Continuum—Short Form (MHC-SF), respectively.

Secondary outcomes included sense of coherence, resilience, mindfulness, and perceived stress, assessed using the Sense of Coherence-13 (SOC-13), Connor–Davidson Resilience Scale (CD-RISC-10), Mindful Attention Awareness Scale (MAAS), and Perceived Stress Scale (PSS-10), respectively.

These outcomes were selected based on their theoretical and empirical relevance to mental health promotion, as well as their consistent use in evaluating the effects of mindfulness-based interventions on both symptom reduction and the enhancement of psychological resources. This selection is aligned with the study hypothesis that mindfulness-based interventions can simultaneously reduce psychological distress and strengthen protective factors associated with positive mental health.

Additionally, sociodemographic, academic, and health-related variables were collected, including prior diagnosis of mental disorders.

All instruments had been previously validated for use in Brazil and demonstrated good internal consistency in the present study (Cronbach’s α ≥ 0.85 across measures).

#### 2.4.1. Primary Outcomes

The GAD-7 assesses the severity of anxiety symptoms over the past two weeks [39]. An example item is “Feeling nervous, anxious, or on edge”. Items are rated on a four-point Likert scale (0–3), with total scores ranging from 0 to 21; higher scores indicate greater anxiety severity. The validated Portuguese version was used [40]. Internal consistency was high (α = 0.873 pre-intervention; α = 0.904 post-intervention).

The PHQ-9 evaluates the presence and severity of depressive symptoms over the past two weeks [41]. An example item is “Feeling down, depressed, or hopeless”. Items are rated on a four-point Likert scale (0–3), yielding total scores from 0 to 27, with higher scores indicating greater symptom severity. The validated Brazilian version was used [42]. Internal consistency was excellent (α = 0.907 pre-intervention; α = 0.908 post-intervention).

The MHC-SF assesses positive mental health, including emotional, social, and psychological well-being [22]. An example item is “During the past month, how often did you feel happy?” Responses are given on a six-point Likert scale (1–6), with total scores ranging from 14 to 84; higher scores indicate better mental health. The validated Brazilian version was used [43]. Internal consistency was high (α = 0.904 pre-intervention; α = 0.928 post-intervention).

#### 2.4.2. Secondary Outcomes

The SOC-13 assesses comprehensibility, manageability, and meaningfulness [19]. An example item is “Do you have the feeling that you are in an unfamiliar situation and don’t know what to do?” Items are rated on a seven-point Likert scale, with some items reverse-scored; total scores range up to 91, with higher scores indicating stronger sense of coherence. The validated Brazilian version was used [44]. Internal consistency was adequate (α = 0.858 pre-intervention; α = 0.864 post-intervention).

The 10-item Connor–Davidson Resilience Scale assesses individuals’ perceived ability to cope with adversity and adapt to change. An example item is “I am able to adapt when changes occur.” Items are rated on a five-point Likert scale (0–4), with total scores ranging from 0 to 40; higher scores indicate greater resilience. The Brazilian validated version (CD-RISC-10BRASIL©) was used [45,46], with authorization from the copyright holders. Internal consistency was high (α = 0.853 pre-intervention; α = 0.884 post-intervention).

The MAAS is a 15-item scale assessing trait mindfulness [14]. An example item is “I find myself doing things without paying attention”. Items are rated on a six-point Likert scale, with higher scores indicating higher levels of mindfulness. The validated Brazilian version was used [47]. Internal consistency was high (α = 0.902 pre-intervention; α = 0.918 post-intervention).

The PSS-10 assesses the degree to which individuals perceive situations in their lives as stressful over the past month [48,49]. An example item is “In the last month, how often have you felt that you were unable to control important things in your life?” Items are rated on a five-point Likert scale (0–4), with total scores ranging from 0 to 40; higher scores indicate greater perceived stress. The validated Brazilian version was used [50]. Internal consistency was high (α = 0.882 pre-intervention; α = 0.905 post-intervention).

### 2.5. Statistical Analysis

Data are presented as mean, standard deviation (SD), standard error (SE), absolute (n), relative frequencies (%) and 95% confidence interval (95% CI). Sample size calculation was based on previous findings [51], assuming 80% power, a significance level of 0.05, and expected differences in PHQ-9 scores between groups. The minimum estimated sample size was 102 participants (51 per group), accounting for a 20% attrition rate. However, recruitment was not restricted, as the intervention was offered as an open course. All analyses followed the intention-to-treat principle [52,53] and adhered to CONSORT recommendations. Internal consistency of the instruments was assessed using Cronbach’s alpha. Baseline group comparability was evaluated using independent *t*-tests for continuous variables and Fisher’s exact test for categorical variables. Besides that, Cohen’s *d* values were calculated between-group at post-intervention.

To assess the potential impact of attrition on study validity, baseline differences between participants who completed the post-intervention assessment and those who discontinued participation were examined using two-way analysis of variance (ANOVA), including study group, dropout status, and group-by-dropout interaction as factors. Age and all primary and secondary outcome measures were evaluated. Results of these analyses are presented in Supplementary Table S1. To assess intervention effects over time and between groups, linear mixed-effects models were employed, accounting for repeated measures within individuals. This approach appropriately handles correlated data and missing observations. All analyses were conducted using SAS software (version 9.4), and figures were generated using R (Version 4.3.1.) Statistical significance was set at α = 0.05.

## 3. Results

**Figure 1:**
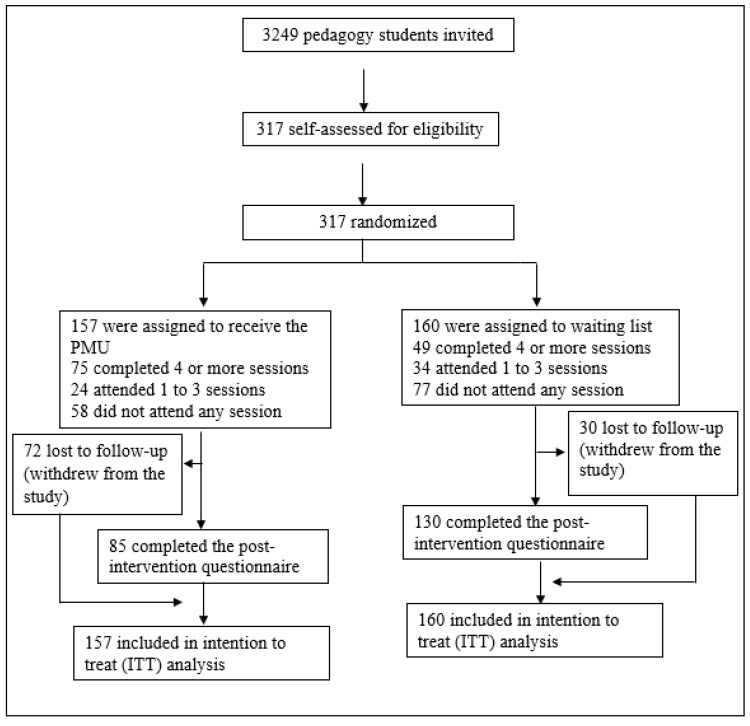
Flowchart of participants throughout the study.

Between 27 May and 20 June 2023, a total of 317 DL pedagogy students were assessed for eligibility and enrolled in the clinical trial. Of these, 157 participants were randomly allocated to the mindfulness-based intervention group, and 160 to the control group (Figure 1).

A total of 135 participants (42.6%) did not attend any intervention session (PMU: *n* = 58; control: *n* = 77). The most frequently reported reasons for non-participation were excessive academic or personal demands and lack of time (*n* = 89), while 46 participants did not provide a reason. Post-intervention data were available for 215 participants. In the intervention group, 85 participants (54.1%) completed the post-intervention assessment, compared with 130 participants (81.3%) in the control group.

Regarding adherence to the intervention, 75 participants (47.8%) attended at least four sessions, while 24 (15.3%) attended between one and three sessions. Among those with lower adherence (<4 sessions), the most commonly reported barriers were scheduling conflicts (*n* = 10) and lack of time (*n* = 9), whereas a smaller number of participants (*n* = 5) reported low affinity with the intervention approach. All randomized participants were included in the analyses according to the intention-to-treat principle [52,53]. All instruments demonstrated good internal consistency at both assessment points (Cronbach’s α > 0.80).

### 3.1. Baseline Characteristics

Baseline sociodemographic, clinical, and outcome characteristics are presented in Table 1. The study groups were comparable across baseline characteristics and outcome measures. Detailed distributions of sociodemographic characteristics are provided in Supplementary Table S2. In addition, no statistically significant differences were observed between groups for any primary or secondary outcome measure at baseline (all *p* > 0.05), indicating successful randomization and baseline comparability (Supplementary Table S3).

Participants’ ages ranged from 19 to 63 years in the PMU group (M = 34.39; SD = 8.79) and from 19 to 60 years in the control group (M = 32.92; SD = 9.72). In both groups, the majority of participants were female, married, employed, had a monthly income of up to seven minimum wages, were pursuing their first undergraduate degree, and resided in the South or Southeast regions of Brazil.

Regarding mental health status, 12.7% (20/157) of participants in the PMU group and 14.4% (23/160) in the control group reported at least one previously diagnosed mental disorder. Most participants reported no prior experience with mindfulness practices.

### 3.2. Primary Outcomes

As shown in Table 2, the mindfulness-based intervention had statistically significant effects on all primary outcomes. Values presented in Table 2 are estimated marginal means and standard errors derived from the linear mixed-effects models, together with adjusted between-group differences, 95% confidence intervals, *p*-values, and standardized effect sizes.

Mental health scores (MHC-SF) increased in the PMU group, while scores in the control group remained relatively stable. The adjusted between-group difference at post-intervention was statistically significant, favoring the intervention. Anxiety symptoms (GAD-7) decreased in both groups; however, the reduction was substantially greater in the PMU group. Similarly, depressive symptoms (PHQ-9) decreased more markedly among participants receiving the intervention than among those in the control group. Detailed estimated marginal means, standard errors, between-group differences, 95% confidence intervals, and standardized effect sizes are presented in Table 2.

Regarding the magnitude of intervention effects, large standardized effect sizes were observed for all primary outcomes. Positive mental health showed a large between-group effect (Cohen’s *d* = 1.08), indicating substantial improvements in overall well-being among participants in the intervention group. Similarly, large effects were observed for reductions in anxiety (*d* = 1.03) and depressive symptoms (*d* = 1.10), with negative values indicating lower symptom scores in the PMU group compared with the control group.

### 3.3. Secondary Outcomes

As shown in Table 3, significant improvements were observed across all secondary outcomes in the PMU group compared with the control group. Participants receiving the intervention demonstrated significant increases in sense of coherence, resilience, and mindfulness, whereas no significant changes were observed in the control group. Perceived stress decreased in both groups; however, the reduction was substantially greater among participants in the PMU group, resulting in a significant between-group difference favoring the intervention. Detailed estimated means, between-group differences, 95% confidence intervals, and standardized effect sizes are presented in Table 3.

Regarding the magnitude of intervention effects, large standardized effect sizes were observed for perceived stress (Cohen’s *d* = 1.08) and sense of coherence (*d* = 0.91). Resilience demonstrated a moderate-to-large effect (*d* = 0.71), while improvements in mindfulness, as measured by the MAAS, were smaller but remained statistically significant, with a small-to-moderate effect size (*d* = 0.43). Overall, these findings indicate that the intervention not only reduced psychological distress but also strengthened important psychological resources associated with mental health and well-being.

While Table 2 and Table 3 present between-group intervention effects, detailed within-group changes are provided in Supplementary Table S4. The distribution of post-intervention scores across all primary and secondary outcomes is presented in Supplementary Figure S1.

### 3.4. Attrition Analysis

Given the relatively high attrition rate, additional analyses were conducted to evaluate potential differential dropout bias [54]. As shown in Supplementary Table S1, no significant baseline differences were observed between participants who completed the study and those who discontinued regarding age, positive mental health, anxiety, depression, perceived stress, sense of coherence, resilience, or mindfulness (all *p* > 0.05). Furthermore, no significant group-by-dropout interactions were detected. These findings suggest that attrition was not systematically associated with baseline participant characteristics or outcome measures and therefore was unlikely to have substantially biased the intervention effect estimates.

## 4. Discussion

This study evaluated the effects of an online mindfulness-based intervention on mental health and psychological resources among Brazilian undergraduate students. The intention-to-treat analysis revealed significant improvements across all primary outcomes, including increased positive mental health and reduced symptoms of anxiety and depression compared with the control group. Significant benefits were also observed in secondary outcomes, with reductions in perceived stress and increases in resilience, sense of coherence, and mindfulness. Together, these findings support the study hypotheses and indicate that the intervention produced meaningful benefits across multiple dimensions of mental health, highlighting its potential as a preventive and health-promoting strategy for university students.

Baseline anxiety and depression scores were consistent with moderate symptom levels and comparable to those reported in Brazilian populations, although higher than those observed in international samples [24,55,56,57]. In contrast, baseline levels of positive mental health were higher than those reported in previous national and international studies [24,56,58]. These differences may reflect cultural variations in the perception and reporting of well-being and psychological distress, which should be considered when interpreting cross-cultural comparisons [59].

The observed improvements in mental health and well-being are consistent with previous studies demonstrating that mindfulness-based interventions enhance well-being while reducing psychological distress [29,51,60,61]. The MHC-SF assesses positive mental health across emotional, psychological, and social dimensions, which are closely associated with psychological resources such as resilience, mindfulness, sense of coherence, social support, and gratitude [43,62]. Together, these findings suggest that mindfulness-based interventions may foster mental health not only by alleviating distress but also by strengthening adaptive psychological resources that contribute to flourishing and resilience.

The reductions in anxiety and depressive symptoms observed in this study are consistent with findings from randomized controlled trials and meta-analyses conducted across diverse university populations and intervention formats [29,51,60]. Similar benefits have been reported in online and web-based mindfulness interventions targeting university students, including programs delivered during the COVID-19 pandemic [63,64,65,66,67,68]. Given the high prevalence of anxiety and depression among university students and their negative impact on academic performance, social functioning, and quality of life, these findings are particularly relevant. The concurrent increase in positive mental health alongside reductions in anxiety and depressive symptoms further supports the dual-continua model, which conceptualizes mental health as more than the absence of mental illness and emphasizes the presence of positive psychological functioning and well-being [62]. Additional support for the transdiagnostic benefits of mindfulness training comes from randomized controlled trials conducted in clinical populations, where mindfulness-based interventions have also produced significant reductions in symptoms of anxiety and depression [69].

Traditional programs such as Mindfulness-Based Stress Reduction (MBSR) and Mindfulness-Based Cognitive Therapy (MBCT) have consistently demonstrated effectiveness in reducing psychological distress while improving emotional regulation and overall psychological functioning [13,70,71,72]. This convergence of evidence highlights the robustness and versatility of mindfulness-based approaches as preventive mental health strategies in higher education settings. Consistent with this broader body of evidence, perceived stress also decreased substantially in the present study, aligning with previous clinical trials demonstrating that mindfulness-based interventions improve stress management across different delivery formats [73,74,75]. Although some reduction was also observed in the control group, this may reflect contextual factors, such as variations in academic workload throughout the semester. Beyond self-reported outcomes, previous studies have shown that mindfulness training may also attenuate biological stress responses, suggesting that reductions in perceived stress may reflect broader psychophysiological adaptations [76].

The intervention also strengthened key psychological resources. While increases in mindfulness were statistically significant, the magnitude of change was smaller compared with other outcomes, suggesting that the benefits of mindfulness-based interventions may extend beyond attentional processes alone [36,77,78]. Mechanisms such as reduced emotional reactivity and improved coping may play a central role in explaining the observed improvements in mental health outcomes [65,79,80]. Resilience also increased significantly, although this construct is complex and may be less sensitive to short-term interventions [23]. Similar findings have been reported in recent clinical trials [34,74], although studies involving healthy populations do not always detect changes in resilience [32,61]. Together, these findings suggest that mindfulness-based interventions may strengthen psychological resources that support adaptation and well-being beyond symptom reduction. Consistent with this view, university-based randomized trials have reported improvements in psychological well-being, personal strengths, and academic retention following mindfulness interventions [35].

The intervention also produced meaningful improvements in sense of coherence, one of the central constructs of the salutogenic model. Sense of coherence reflects the extent to which individuals perceive life as comprehensible, manageable, and meaningful, influencing how they interpret and respond to stressful experiences [19]. From this perspective, individuals with a stronger sense of coherence are better able to mobilize available resources, cope effectively with challenges, and maintain psychological well-being despite adversity. The observed improvement is particularly relevant because sense of coherence has consistently been associated with better mental health outcomes, resilience, and psychological well-being [22,24,25,56]. Recent evidence further suggests that stronger sense of coherence is associated with lower depressive symptom severity and better overall mental health outcomes [80]. Although still relatively underexplored in mindfulness research, emerging evidence suggests that mindfulness-based interventions may strengthen salutogenic resources by fostering greater awareness, acceptance, and adaptive responses to life stressors [18,20]. These findings support the potential of mindfulness-based interventions not only to reduce psychological distress but also to strengthen broader psychological resources that promote long-term adaptation and well-being [21].

The intervention evaluated in the present study followed the traditional eight-week structure of the Mindfulness-Based Health Promotion (MBHP) program, which is consistent with established mindfulness-based interventions derived from MBSR. This format was selected to preserve fidelity to the original protocol and because it remains the most widely investigated and empirically supported format in mindfulness research. Nevertheless, emerging evidence suggests that shorter mindfulness interventions may also produce meaningful improvements in mental health outcomes. For example, studies have reported beneficial effects for abbreviated programs lasting four to five weeks, as well as for brief interventions delivered over a few consecutive days [32,66,81,82]. Some findings suggest that shorter formats may achieve improvements comparable to standard-length programs for certain outcomes, whereas longer interventions may provide additional benefits for more complex or sustained psychological changes [32,83].

The relatively low adherence and high attrition observed in this trial provide important information regarding the feasibility of implementing eight-week online mindfulness programs. Although the duration of the intervention was chosen to preserve fidelity to the original MBHP protocol and to support the development of both symptom-related and salutogenic outcomes, competing academic, professional, and family responsibilities may have limited participant engagement. This interpretation is consistent with previous studies reporting challenges in maintaining adherence to mindfulness-based interventions among students [84]. For this reason, future research should directly compare standard and abbreviated formats to determine whether shorter interventions can achieve similar benefits while improving adherence and scalability in university settings.

Importantly, the online format adopted in this study did not appear to compromise effectiveness. Previous research indicates that digital and remotely delivered mindfulness interventions can produce outcomes comparable to in-person formats [33,36,63,64,75,85], while substantially increasing accessibility and scalability. These characteristics are particularly relevant in contexts with limited access to mental health services, such as low- and middle-income settings.

From a public health perspective, these findings are particularly relevant given the high prevalence of anxiety, depression, and psychological distress among university students and the limited availability of support services in many higher education institutions. Online mindfulness-based interventions may represent a scalable and cost-effective strategy for expanding access to mental health promotion, not only by reducing psychological distress but also by strengthening psychological resources associated with long-term well-being and adaptation. Integration with existing student support services, flexible delivery formats, and strategies to enhance engagement may facilitate implementation and maximize impact. The ability to deliver such programs remotely may be especially valuable in low- and middle-income countries, where resource constraints frequently limit the availability of more intensive interventions.

This study has several important strengths. First, the randomized controlled design enhances internal validity and supports causal inference regarding the observed intervention effects. Second, the study included a large sample of undergraduate students and employed validated instruments to assess a broad range of mental health outcomes and psychological resources. Third, the use of an intention-to-treat analytical approach and linear mixed-effects models strengthened the robustness of the findings by incorporating all available data and appropriately handling missing observations. Additionally, adverse effects were systematically monitored throughout the intervention, providing a more comprehensive evaluation of its safety and feasibility. To our knowledge, this is one of the largest randomized controlled trials evaluating an online mindfulness-based intervention among undergraduate students in Brazil.

Several limitations should also be acknowledged. Participation was voluntary, which may have introduced selection bias by attracting students with greater interest in mindfulness or mental health promotion. The use of self-reported measures and the absence of participant blinding may have influenced responses through reporting and expectancy biases. The intervention was delivered by a single instructor, and the sample consisted predominantly of female participants, with a relatively high proportion of married students, which may limit the generalizability of the findings. The use of a passive control group, which does not fully control for expectancy or attention effects, and the absence of data regarding home mindfulness practice should also be considered when interpreting the findings.

Furthermore, adherence was lower than anticipated and attrition rates were substantial, particularly within the intervention group. However, additional analyses revealed homogeneity between completers and non-completers across the primary and secondary outcome measures, reducing concerns regarding differential dropout bias. Beyond participant attrition, an additional limitation concerns the assessment of intervention adherence. Although attendance at live sessions was systematically recorded, participants who missed a session were granted access to a recorded version. Because access to and viewing of these recordings were not monitored, actual exposure to intervention content may have been underestimated. Consequently, some participants classified as non-attenders or low attenders may have engaged with the intervention through recorded sessions. This limitation restricted the interpretation of adherence rates and may have influenced estimates of participant engagement with the program. Nevertheless, participant losses may have reduced statistical precision and highlight the importance of developing strategies to improve engagement and retention in future online mindfulness programs.

Finally, although the study included participants with self-reported psychiatric diagnoses, it was not designed or powered to conduct subgroup analyses examining whether intervention effects differed according to clinical status. Future research should investigate potential differences in response to mindfulness-based interventions among students with and without mental health conditions.

Despite these limitations, the study provides a rigorous and ecologically valid evaluation of an accessible, scalable, and low-cost intervention. The online delivery format enhances its potential applicability in real-world educational settings and supports its integration into institutional strategies aimed at promoting student mental health and psychological well-being.

Overall, the findings provide further evidence that online mindfulness-based interventions can effectively promote mental health and strengthen psychological resources among university students. Beyond reducing symptoms of anxiety, depression, and perceived stress, the intervention enhanced positive mental health, resilience, sense of coherence, and mindfulness, supporting a broader health-promotion perspective. Given the high prevalence of psychological distress among undergraduate students and the limited availability of mental health services in many educational settings, scalable and evidence-based interventions such as the present program may represent a valuable strategy for expanding access to preventive mental health care and supporting student well-being at the population level.

## 5. Conclusions

This study demonstrated that an online mindfulness-based intervention was associated with significant improvements in mental health among undergraduate students. Participants in the intervention group showed increased positive mental health, resilience, sense of coherence, and mindfulness, together with reductions in symptoms of anxiety, depression, and perceived stress.

These findings contribute to the growing evidence supporting mindfulness-based interventions as a promising approach not only for reducing psychological distress but also for strengthening salutogenic resources that promote well-being, adaptation, and positive functioning. By simultaneously improving both psychopathological outcomes and positive indicators of mental health, the intervention aligns with contemporary perspectives that conceptualize mental health as more than the absence of mental illness.

Given the high prevalence of psychological distress among undergraduate students and the limited availability of support services in many educational settings, online mindfulness-based programs may represent a valuable and scalable strategy for expanding access to preventive mental health care. However, challenges related to participant engagement and long-term implementation remain important considerations for future research and practice. Future studies should investigate strategies to improve adherence, retention, and long-term sustainability, as well as examine the mechanisms through which mindfulness-based interventions influence both psychological symptoms and salutogenic resources.

## Figures and Tables

**Table 1:** Baseline sociodemographic, clinical, and outcome characteristics of participants by study group.

Baseline Characteristics	PMU (*n* = 157)	Control (*n* = 160)	*p*-Value
Age, mean (SD)	34.84 (8.96)	32.97 (10.38)	0.088
Female, *n* (%)	150 (95.54)	152 (95.0)	0.691
Married/common-law marriage, *n* (%)	101 (64.33)	85 (53.13)	0.083
Paid work, *n* (%)	114 (72.61)	116 (72.5)	1.000
Monthly income ≤ 7 MW, *n* (%)	151 (96.18)	151 (94.38)	0.342
Incomplete higher education, *n* (%)	128 (81.53)	124 (77.5)	0.563
Previous mindfulness practice, *n* (%)	14 (8.92)	9 (5.63)	0.286
Psychiatric diagnosis, *n* (%)	20 (12.74)	23 (14.38)	0.744
Region, *n* (%)			0.172
Midwest	8 (5.1)	20 (12.5)	
Northeast	27 (17.2)	20 (12.5)	
North	8 (5.1)	9 (5.63)	
Southeast	66 (42.04)	62 (38.75)	
South	48 (30.57)	49 (30.63)	
Primary outcomes, mean (SD)			
MHC-SF	57.66 (13.4)	54.88 (13.83)	0.064
GAD-7	10.17 (5.61)	10.28 (5.57)	0.853
PHQ-9	12.12 (7.54)	12.14 (7.48)	0.978
Secondary outcomes, mean (SD)			
PSS-10	20.45 (7.15)	21.71 (6.98)	0.108
SOC-13	55.97 (16.42)	55.06 (14.88)	0.601
CD-RISC-10	22.21 (7.94)	22.64 (7.49)	0.629

Data are presented as *n* (%) unless otherwise specified. Continuous variables are presented as mean and standard deviation. Baseline outcome scores represent observed, unadjusted values before the intervention. *p*-values for baseline outcome comparisons were obtained from between-group comparisons at pre-intervention. PMU = Mindfulness-Based Program for Undergraduate Students; MW = minimum wages; MHC-SF = Mental Health Continuum–Short Form (positive mental health); GAD-7 = Generalized Anxiety Disorder-7 (anxiety); PHQ-9 = Patient Health Questionnaire-9 (depression); PSS-10 = Perceived Stress Scale-10 (stress); SOC-13 = Sense of Coherence Scale-13 (sense of coherence); CD-RISC-10 = Connor-Davidson Resilience Scale-10 (resilience); MAAS = Mindful Attention Awareness Scale (mindfulness). No statistically significant differences were observed between groups at baseline (all *p* > 0.05).

**Table 2:** Effects of the online mindfulness-based intervention on primary outcomes (intention-to-treat analysis).

Outcome	Mean (SE)				Between-Group Difference (95% CI)	Cohen’s *d*	*p*-Value
	PMU		Control				
	Pre	Post	Pre	Post			
MHC-SF	57.66 (1.06)	63.71 (1.30)	54.88 (1.05)	55.15 (1.12)	8.57 (5.19 to 11.94)	1.08	<0.001
GAD-7	10.17 (0.44)	5.68 (0.55)	10.28 (0.44)	9.34 (0.47)	−3.66 (−5.09 to −2.23)	−1.03	<0.001
PHQ-9	12.12 (0.57)	6.58 (0.70)	12.14 (0.57)	11.19 (0.60)	−4.61 (−6.43 to −2.80)	−1.10	<0.001

Values are estimated marginal means (SE) obtained from linear mixed-effects models. Between-group differences represent adjusted post-intervention differences between the PMU and control groups. Cohen’s *d* values represent standardized adjusted between-group effect sizes at post-intervention. Positive values indicate higher scores in the PMU group compared with the control group, whereas negative values indicate lower scores in the PMU group. For symptom outcomes, negative values favor the intervention. 95% CI = 95% confidence interval. PMU = Mindfulness-Based Program for Undergraduate Students. Pre = pre-intervention. Post = post-intervention. MHC-SF = Mental Health Continuum–Short Form (positive mental health). GAD-7 = Generalized Anxiety Disorder-7 (anxiety). PHQ-9 = Patient Health Questionnaire-9 (depression).

**Table 3:** Effects of the online mindfulness-based intervention on secondary outcomes (intention-to-treat analysis).

Outcome	Mean (SE)				Between-Group Difference (95% CI)	Cohen’s *d*	*p*-Value
	PMU		Control				
	Pre	Post	Pre	Post			
PSS-10	20.45 (0.56)	16.02 (0.67)	21.71 (0.55)	20.38 (0.58)	−4.36 (−6.12 to −2.60)	−1.08	<0.001
SOC-13	55.97 (1.24)	63.51 (1.48)	55.06 (1.22)	55.70 (1.30)	7.81 (3.92 to 11.69)	0.91	<0.001
CD-RISC-10	22.21 (0.63)	25.70 (0.76)	22.64 (0.62)	22.40 (0.66)	3.30 (1.31 to 5.30)	0.71	0.001
MAAS	3.79 (0.09)	4.01 (0.11)	3.73 (0.09)	3.73 (0.09)	0.28 (0.001 to 0.56)	0.43	0.049

Values are estimated marginal means (SE) obtained from linear mixed-effects models. Between-group differences represent adjusted post-intervention differences between the PMU and control groups. Cohen’s *d* values represent standardized adjusted between-group effect sizes at post-intervention. Positive values indicate higher scores in the PMU group compared with the control group, whereas negative values indicate lower scores in the PMU group. For symptom outcomes, negative values favor the intervention. 95% CI = 95% confidence interval. PMU = Mindfulness-Based Program for Undergraduate Students. Pre = pre-intervention. Post = post-intervention. PSS-10 = Perceived Stress Scale-10 (stress). SOC-13 = Sense of Coherence Scale-13 (sense of coherence). CD-RISC-10 = Connor-Davidson Resilience Scale-10 (resilience). MAAS = Mindful Attention Awareness Scale (mindfulness).

## Data Availability

The datasets generated and analyzed during the current study are not publicly available due to ethical and privacy restrictions related to participant confidentiality. However, de-identified data may be available from the corresponding author upon reasonable request.

## Supplementary Materials

The following supporting information can be downloaded at https://www.mdpi.com/article/10.3390/ijerph23070853/s1, Table S1: Baseline comparison between completers and dropouts; Table S2: Detailed baseline sociodemographic characteristics of participants by study group; Table S3: Baseline comparison of primary and secondary outcome measures between study groups; Table S4: Within-group changes from baseline to post-intervention for primary and secondary outcomes based on intention-to-treat analyses using linear mixed-effects models; Figure S1: Distribution of post-intervention scores according to study group; File S1: CONSORT 2025 checklist. Ref. [86] is cited in the Supplementary Materials.

## Abbreviations

The following abbreviations are used in this manuscript:

ReBEC	Brazilian Registry of Clinical Trials (ReBEC)
CD-RISC-10	Connor–Davidson Resilience Scale (instrument)
CONSORT	Consolidated Standards of Reporting Trials
GAD-7	Generalized Anxiety Disorder-7 (instrument)
MAAS	Mindful Attention Awareness Scale (instrument)
MBCT	Mindfulness-Based Cognitive Therapy
MBHP	Mindfulness-Based Health Promotion
MBIs	Mindfulness-Based Interventions
MBSR	Mindfulness-Based Stress Reduction
MHC-SF	Mental Health Continuum—Short Form (instrument)
DL	Distance Learning
PHQ-9	Patient Health Questionnaire-9 (instrument)
PMU	Mindfulness-Based Program for Undergraduate Students
PSS-10	Perceived Stress Scale ((instrument)
SOC-13	Sense of Coherence-13 (instrument)
WHO	World Health Organization
